# Verification of the effect of acquisition time for SwiftScan on quantitative bone single-photon emission computed tomography using an anthropomorphic phantom

**DOI:** 10.1186/s40658-022-00477-9

**Published:** 2022-07-30

**Authors:** Takuro Shiiba, Yuya Sekikawa, Shinji Tateoka, Nobutaka Shinohara, Yuuki Inoue, Yasuyoshi Kuroiwa, Takashi Tanaka, Yasushi Kihara, Takuroh Imamura

**Affiliations:** 1 Department of Molecular Imaging, School of Medical Sciences, Fujita Health University, 1-98, Dengakubo, Kutsukake-cho, Aichi 470-1192 Toyoake, Japan; 2 Department of Radiological Technology, Faculty of Fukuoka Medical Technology, Teikyo University, 6-22 Misakimachi, Omuta-shi, Fukuoka 836-8505 Japan; 3 Department of Radiological Technology, Koga General Hospital, 1749-1 Sudaki, Ikeuchi-cho, Miyazaki-shi, Miyazaki, 880-0041 Japan; 4 Department of Pathology, Faculty of Medicine, University of Miyazaki, 5200 Kihara, Kiyotake, Miyazaki, 889-1692 Japan; 5 Department of Radiology, Koga General Hospital, 1749-1 Sudaki, Ikeuchi-cho, Miyazaki-shi, Miyazaki, 880-0041 Japan; 6 Department of Internal Medicine, Koga General Hospital, 1749-1 Sudaki, Ikeuchi-cho, Miyazaki-shi, Miyazaki, 880-0041 Japan

**Keywords:** SwiftScan, Single-photon emission computed tomography, Bone, Quantification, Acquisition time

## Abstract

**Background:**

SwiftScan single-photon emission computed tomography (SPECT) is a recently released scanning technique with data acquired when the detector is stationary and when it moves from one view to the next. The influence of scan time for using SwiftScan on quantitative bone SPECT remains unclear. This study aimed to clarify the effect of the scan time for SwiftScan SPECT on the image quality and quantification of bone SPECT compared to step and shoot mode (SSM) using ^99m^Tc-filled anthropomorphic phantom (SIM^2^ bone phantom).

**Materials and methods:**

Phantom SPECT/computed tomography (CT) images were acquired using Discovery NM/CT 860 (GE Healthcare) with a low-energy high-resolution sensitivity collimator. We used the fixed parameters (subsets 10 and iterations 5) for reconstruction. The coefficient of variation (CV), contrast-to-noise ratio (CNR), full width at half maximum (FWHM), and quantitative value of SwiftScan SPECT and SSM were compared at various acquisition times (5, 7, 17, and 32 min).

**Results:**

In the short-time scan (< 7 min), the CV and CNR of SwiftScan SPECT were better than those of SSM, whereas in the longtime scan (> 17 min), the CV and CNR of SwiftScan SPECT were similar to those of SSM. The FWHMs for SwiftScan SPECT (13.6–14.8 mm) and SSM (13.5–14.4 mm) were similar. The mean absolute errors of quantitative values at 5, 7, 17, and 32 min were 38.8, 38.4, 48.8, and 48.1, respectively, for SwiftScan SPECT and 41.8, 40.8%, 47.2, and 49.8, respectively, for SSM.

**Conclusions:**

SwiftScan on quantitative bone SPECT provides improved image quality in the short-time scan with quantification similar to or better than SSM. Therefore, in clinical settings, using SwiftScan SPECT instead of the SSM scan protocol in the short-time scan might provide higher-quality diagnostic images than SSM. Our results could provide vital information on the use of SwiftScan SPECT.

## Background

Bone scintigraphy is used to detect bone metastases, such as in prostate cancer [1] and breast cancer [2]. In prostate cancer, bone scintigraphy exhibits a high sensitivity to bone metastasis, making it one of the most valuable methods for the detection of bone metastasis [1]. To determine the cancer stage and effective treatment strategies as well as disease prognosis, it is essential to be aware of the presence of bone metastasis [3].

In bone scintigraphy, single-photon emission computed tomography (SPECT) is used in addition to whole-body imaging to facilitate three-dimensional positioning and improve the detection rate of lesions. Recently, systems combining SPECT with computed tomography (CT) have become widely available, and improved accuracy of bone lesion diagnosis through SPECT/CT imaging has been reported [4–7].

Quantification of ^99m^Tc bone SPECT/CT is becoming feasible as a diagnostic tool and as a means of monitoring treatment efficacy [8]. Phantom and clinical studies have reported that the quantitative accuracy of ^99m^Tc-based SPECT imaging is within ± 10% [9]. However, for bone SPECT quantification to become a standard clinical diagnostic method, the accumulation of more reliable quantitative data is needed. The accuracy of bone SPECT quantification is affected by the acquisition method, resolution, reconstruction method, and cross-calibration method of the device [10–13]. Acquisition methods, such as the step and shoot mode (SSM) and continuous mode (CM) [14], are commonly used for SPECT. SwiftScan (GE Healthcare, Milwaukee, WI, USA) [15–18] SPECT is a recently released scanning technique with data acquired when the detector is stationary and when it moves from one view to the next [19]. Thibault et al. [17] reported that the GE-developed SwiftScan with low-energy high-resolution sensitivity (LEHRS) collimator exhibited improved sensitivity compared to that of LEHR collimator. Shibutani et al. [18] reported that the SwiftScan planar and SPECT with LEHRS collimator have high sensitivity while maintaining the spatial resolution compared with the conventional SPECT system. Several clinical studies have shown the potential of SwiftScan SPECT in reducing acquisition time. Bailly et al. [16] and Picone et al. [20] showed the potential for a 25% reduction in acquisition time without degradation of image quality and quantitation in dopamine transporter and bone, respectively. However, to our knowledge, no studies so far have investigated the effect of acquisition time in SwiftScan using a phantom in quantitative bone SPECT. This study therefore aimed to clarify the effect of acquisition time for SwiftScan on the image quality and quantitative value of quantitative bone SPECT in comparison with SSM.

## Methods

### Phantom

**Fig. 1:**
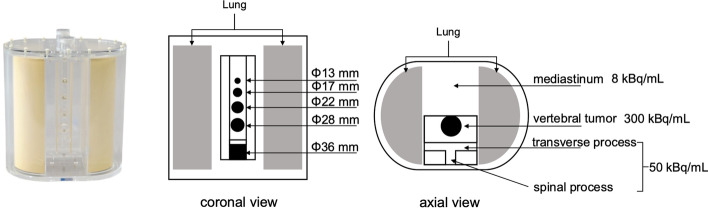
Overview of the SIM^2^ phantom and enclosed radioactivity concentration

The SIM^2^ Bone Phantom (Kyoto Kagaku, Kyoto, Japan) [15, 21] was used. This phantom can reproduce tumor bone area in the vertebral body with four different diameters (13, 17, 22, and 28 mm) using the whole vertebral body as reference (diameter and length of 36 mm and 35 mm, respectively) (Fig. 1). The ^99m^Tc activity concentrations of the normal vertebras, tumor, and mediastinum of the phantom filled with ^99m^Tc were 50, 300, and 8 kBq/mL, respectively [15]. The lung insert was not filled with radioactive material.

### Acquisition and reconstruction of SPECT/CT imaging

Phantom SPECT/CT images were acquired using Discovery NM/CT 860 (GE Healthcare) with a LEHRS collimator. For the dual-energy window scatter correction (SC) method (scatter weighting factor k was 1.10), the primary and scatter window acquisitions were 140 keV ± 10% and 120 keV ± 5%, respectively. The matrix size for the acquisition was 128 × 128, the magnification ratio was 1.0, the pixel size was 4.42 mm, and the projection number was 60 (step of 6°); automatic proximity was used, and the images were acquired with SwiftScan and SSM. The acquisition time was 6, 10, 30, and 60 s/view for SSM. In SwiftScan SPECT, counts are also acquired during 6° step detector rotation; 0–3°counts were added to the previous position view and 3–6°counts were integrated into the next position view. Thus, acquisition time per projection for SwiftScan was added to the detector rotation time (approximately 4 s per view) to that of SSM. Here, the scan time was defined as the start of projection data acquisition to its end. The scan times for SwiftScan SPECT and SSM were 5 min 4 s, 7 min 4 s, 17 min 4 s, and 32 min 4 s. The scans were performed in increasing order of scan times, and the counts and quantification values obtained were decay-corrected by the elapsed time from the start time of the first scan. CT images for attenuation correction (AC) were acquired at 120 kV and 30 mA with a 512 × 512 matrix, 1.675 pitch, and 1.0 s rotation and reconstructed at a 1.25 mm slice thickness with adaptive statistical iterative reconstruction (SS80 Slice 80%). All acquired projection images were reconstructed using a three-dimensional iterative ordered subset expectation maximization algorithm considering the CT-based AC, dual-energy SC, and resolution correction (Evolution for bone, GE Healthcare) without a noise reduction filter. The various combinations of the number of subsets and iterations were 10 (fixed) and 1–10, respectively. After reconstruction, a quantitative analysis software (Q.Volumetrix, GE Healthcare) was used to automatically resample both the CT and SPECT images to a voxel size of 2.21 × 2.21 × 2.21 mm^3^ prior to setting the volume of interest (VOI). This software calculates quantitative values using the planar sensitivity-based calibration method [13, 22, 23]. In this study, the Q.Volumetrix used was designed to calculate quantitative values without noise reduction filters. Therefore, all SPECT image quality evaluations were performed using SPECT images without noise reduction filters.

### System planar sensitivity

The system planar sensitivity was measured using the automatic mode provided by the manufacturer (GE Healthcare). A plastic petri dish with ^99m^Tc solution (124.4 MBq) was placed on styrene foam (10 cm thickness) at the collimator surface, and the image was acquired from the anterior and posterior views. The primary and scatter window acquisitions were 140 keV ± 10% and 120 keV ± 5%, respectively. The system planar sensitivity was calculated using the following formula:

1 $${\text{system planar sensitivity }}\left( {{\text{cps}}/{\text{MBq}}} \right) = \left( {C_{{\text{p}}} {-}kC_{{\text{s}}} } \right)/{\text{AT}}$$

where *A* is the decay-corrected activity at the start time of acquisition, *T* is the acquisition time, *k* is the scatter weighting factor, and *C*_p_ and *C*_s_ are the averages of the total counts in the anterior and posterior images for the primary and scatter windows, respectively. The measured system planar sensitivity was 88.4 cps/MBq.

### Convergence of quantitative values

To determine the optimal number of iterations, the convergence of 5- and 32-min acquisition was assessed. Based on the CT images, sphere-shaped VOIs were drawn at the center of each tumor bone area (28-mm hot sphere). The sizes of the VOIs were 80% of that of each hot sphere. The measured maximum and mean radioactivity concentrations (MBq/mL) were obtained using Q.Volumetrix.

### Evaluation of count statistics and image quality

**Fig. 2:**
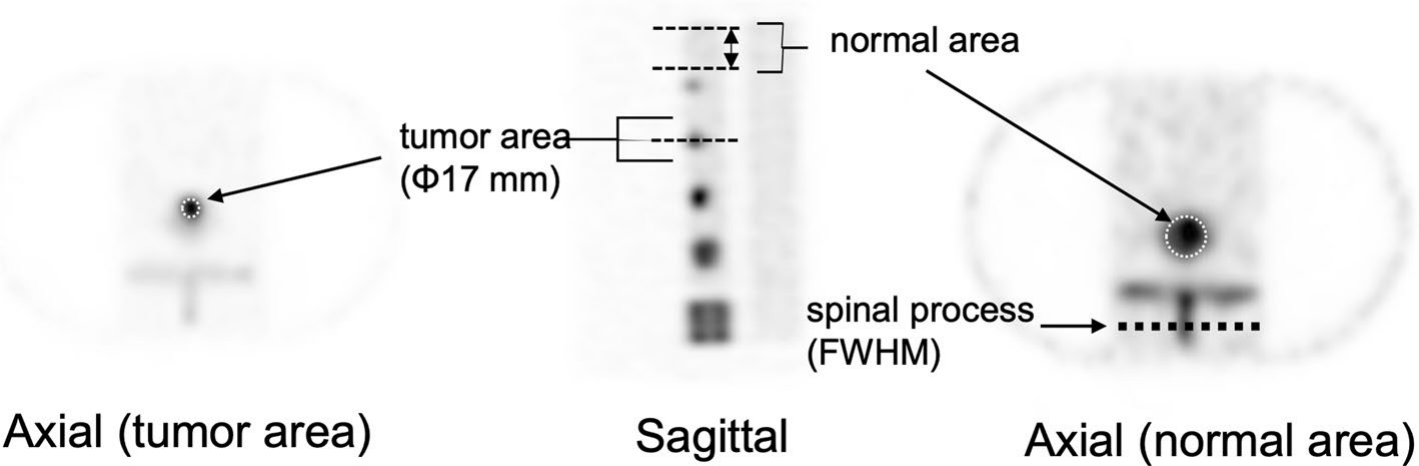
Settings of the region of interest for evaluating image qualities

SPECT images obtained from each acquisition method were compared in terms of normal bone coefficient of variation (CV), contrast-to-noise ratio (CNR), full width at half maximum (FWHM), and quantitative value. For measuring image quality indices, a circular region of interest (ROI) with 80% of the diameter was set in five slices (Fig. 2), including the normal bone portion of the axial image. Similarly, ROIs were set in a slice with the largest diameter of the 17-mm-diameter tumor area. ROI settings were based on CT images. The mean count and standard deviation were obtained from the ROIs of tumor and normal areas. The CV was calculated using the following formula:

2 $${\text{CV}} = \frac{{C_{{{\text{SD}}}} }}{{C_{{{\text{normal}}}} }}$$

where *C*_normal_ and *C*_SD_ represent the mean count and standard deviation of the normal bone area, respectively.

The CNR was calculated using the following formula:

3 $${\text{CNR}} = \frac{{C_{{{\text{tumor}}}} - C_{{{\text{normal}}}} }}{{C_{{{\text{SD}}}} }}$$

where *C*_tumor_ is the mean count of the tumor area.

The effect of scan time on the count statistics of the tumor bone areas was investigated under the optimum number of iterations. Further, the spinous process line profile curves were obtained from five slices of the phantom (Fig. 2), and the FWHM was averaged from those slices using the Prominence processor version 3.1 software [23]

### Accuracy of quantification

Based on the CT images, sphere-shaped VOIs were drawn at the center of each tumor bone area (13-, 17-, 22-, 28-, and 36-mm hot spheres). The sizes of the VOIs were 80% of that of each hot sphere. The measured mean radioactivity concentration (MBq/mL) was obtained using Q.Volumetrix. The error between the true and measured radioactivity concentrations at each tumor bone area was calculated using the following formula.

4 $${\text{Error }}\left( \% \right) = \frac{{\left( {A_{{{\text{True}}}} {-}A_{{{\text{SPECT}}}} } \right) }}{{A_{{{\text{True}}}} }} \times 100$$

where *A*_True_ represents the radioactivity concentration in the tumor bone area of the phantom and *A*_SPECT_ represents the mean radioactivity concentration in the tumor bone area obtained from the SPECT image. Here, *A*_True_ was 300 kBq/mL.

In addition, to evaluate overall quantitative accuracy, the mean absolute error (MAE) of the measured radioactivity concentration for each examination time of SSM and SwiftScan SPECT was calculated.

5 $${\text{MAE}} = \frac{1}{n}\mathop \sum \limits_{i = 13,17,22,28,36}^{{\text{n}}} \left| {A_{{{\text{True}}}} - A_{{i,{\text{SPECT}}}} } \right|$$

where *n* represents the number of tumor bone regions and *A*_*i*,SPECT_ represents measured mean radioactivity concentration in tumor bone sphere size of *i* mm.

## Results

### Convergence of quantitative values

**Fig. 3:**
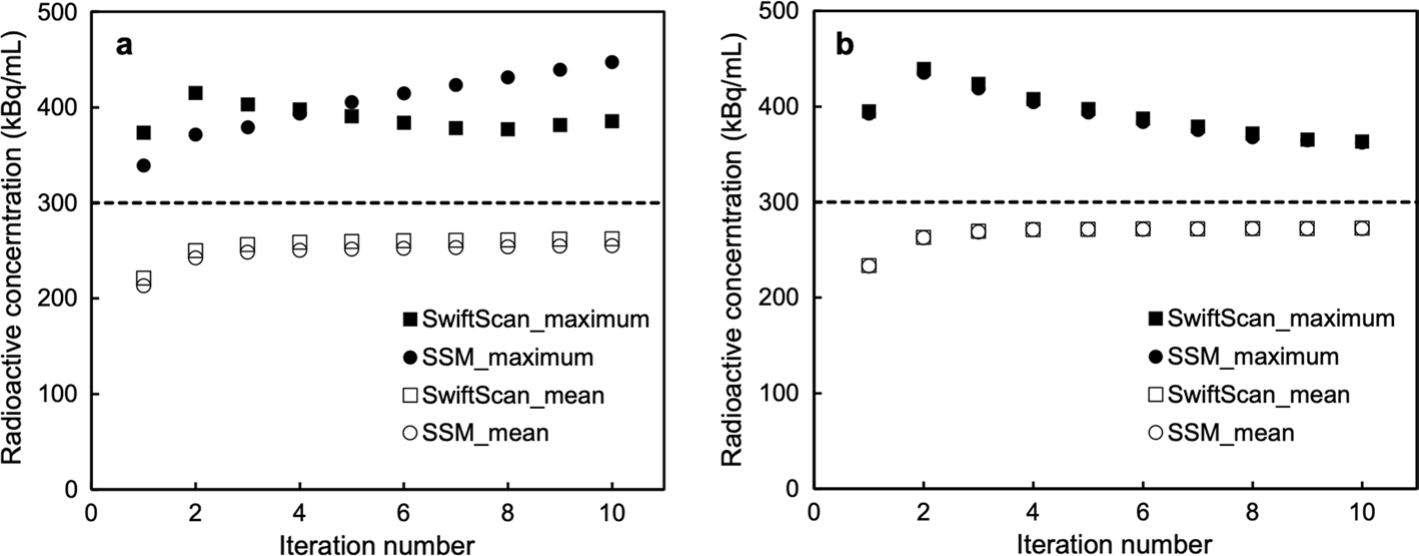
Effects of increasing the number of iterations of reconstruction (with 10 subsets). Effects on **a** short-time (5 min) acquisitions and **b** longtime (32 min) acquisitions. The filled and unfilled squares indicate the maximum and mean radioactivity concentrations of SwiftScan SPECT, respectively. The filled and unfilled circles indicate the maximum and mean radioactivity concentrations of step and shoot mode (SSM), respectively. The dashed line indicates the actual concentration of radioactivity

Figure 3a, b shows the variation in the concentration of radioactivity when the number of iterations was varied. The maximum radioactivity of the SSM at an acquisition time of 5 min increased with an increasing number of iterations. On the other hand, the maximum radioactivity of SwiftScan SPECT tended to decrease more than iteration 2. The mean radioactivity concentration showed that SwiftScan was slightly closer to the actual concentration of radioactivity (dashed line). For both acquisition methods, the average radioactivity concentration converged at five iterations (Fig. 3a). In the 32-min acquisition (Fig. 3b), the maximum radioactivity concentration for SSM and SwiftScan SPECT was close to the actual radioactivity. The mean radioactivity concentrations were similar for both and converged at iteration 5. Based on these results, the iteration was determined to be 5.

### Image quality

#### CV and CNR

**Fig. 4:**
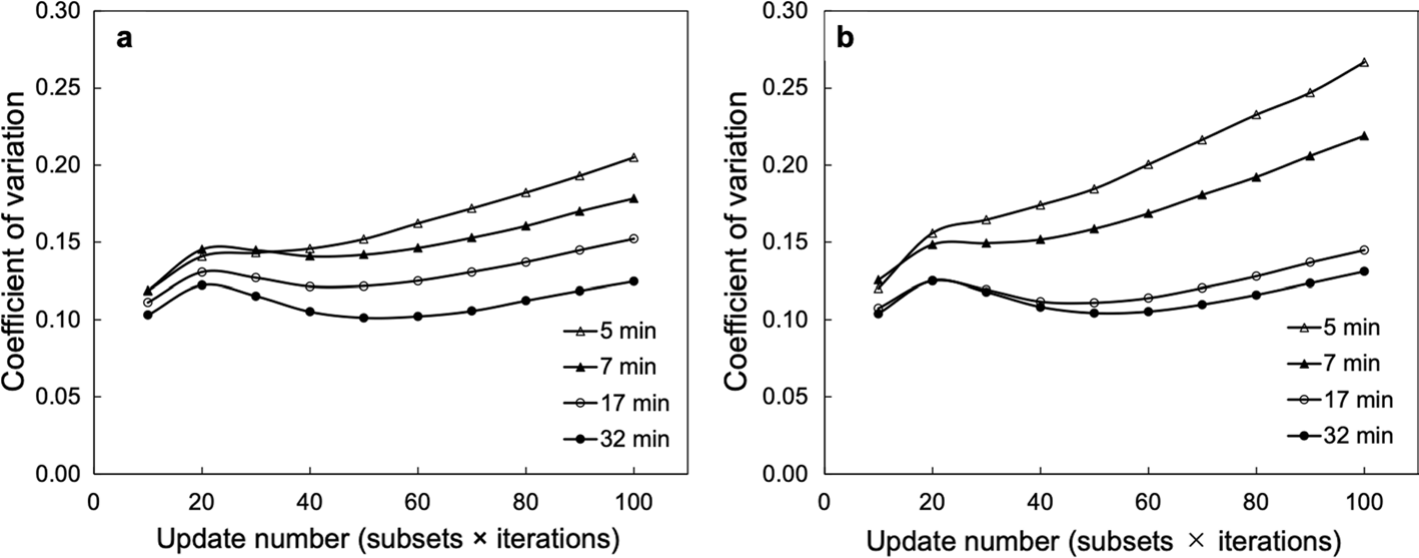
Relationship between update number and coefficient of variation (CV). CV of various acquisition times using **a** SwiftScan SPECT and **b** step and shoot mode (SSM). The update number is the product of the subset and iterations

Figure 4a, b shows the CV of SwiftScan SPECT and SSM, respectively, at different iterations. The CVs of short-time acquisitions in SwiftScan SPECT (Fig. 4a) and SSM (Fig. 4b) were higher than those of longtime acquisitions. Both acquisition methods showed a monotonous increase in the number of iterations from approximately 60. The difference in the CVs of SSM between the short-time and longtime acquisition increased with an increasing number of iterations (Fig. 4b).

**Fig. 5:**
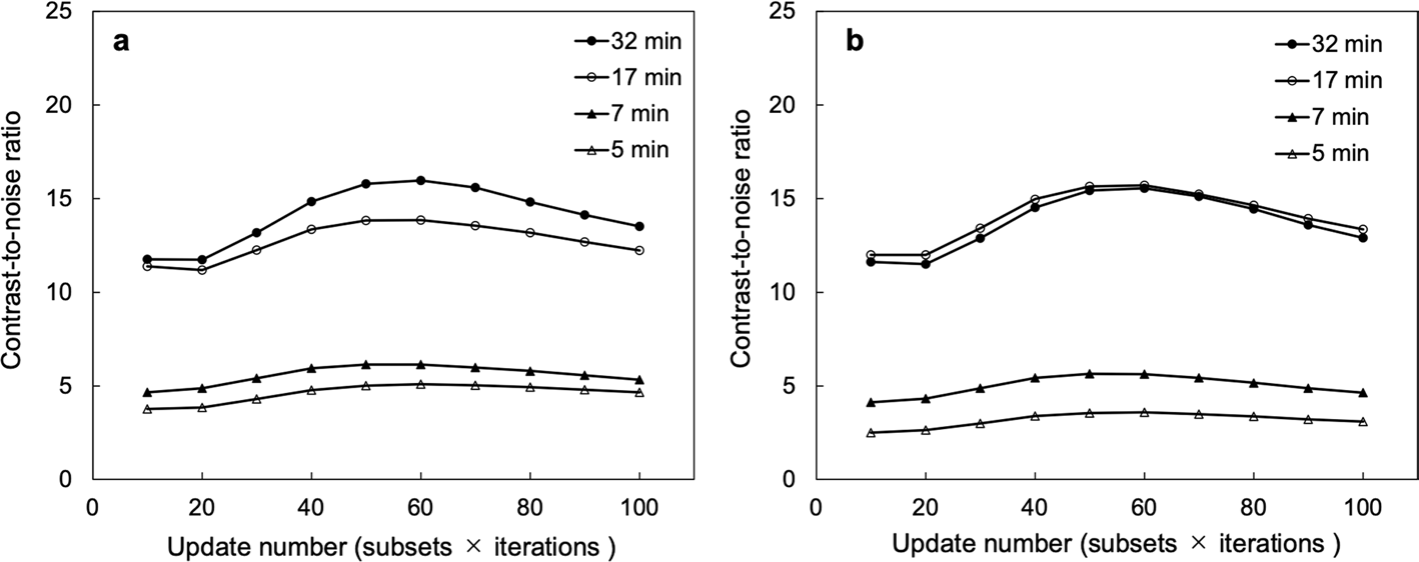
Relationship between update number and contrast-to-noise ratio (CNR). CNR of various acquisition times using **a** SwiftScan SPECT and **b** step and shoot mode (SSM). The update number is the product of the subset and the iterations

Figure 5a, b shows the changes in CNR of SwiftScan SPECT and SSM, respectively, at different iterations. The CNR of both acquisition methods increased with an increasing number of iterations, reaching its highest value at the fifth iteration and tending to decrease subsequently. In SSM, the CNRs for the 17- and 32-min acquisitions were similar (Fig. 5b).

The CV and CNR for SwiftScan and SSM tended to improve with increasing scan times (Table 1). The CV of SSM was higher than that of SwiftScan SPECT in the short-time scan; however, it was similar to that of SwiftScan SPECT in the longtime scan. The CNR of SwiftScan SPECT was higher than that of SSM in the short-time scan. However, there was no consistent trend between SwiftScan SPECT and SSM for longtime scans.

**Table 1:** Comparison of image quality indices and quantitative accuracy between SSM and SwiftScan SPECT

Scan time (min)	CV		CNR		FWHM (mm)		MAE	
	SwiftScan	SSM	SwiftScan	SSM	SwiftScan	SSM	SwiftScan	SSM
5	0.15	0.18	5.01	3.55	14.7 ± 1.9	13.5 ± 1.6	38.8	41.8
7	0.14	0.16	6.13	5.65	14.8 ± 1.7	14.4 ± 1.8	38.4	40.8
17	0.12	0.11	13.8	15.7	13.6 ± 1.2	13.5 ± 1.3	48.8	47.2
32	0.10	0.10	15.8	15.4	14.4 ± 0.6	14.2 ± 0.6	48.1	49.8

*SSM* step and shoot, *CV* coefficient of variation, *CNR* contrast-to-noise ratio, *FWHM* full width at half maximum, *MAE* mean absolute error

### Count statistics

**Fig. 6:**
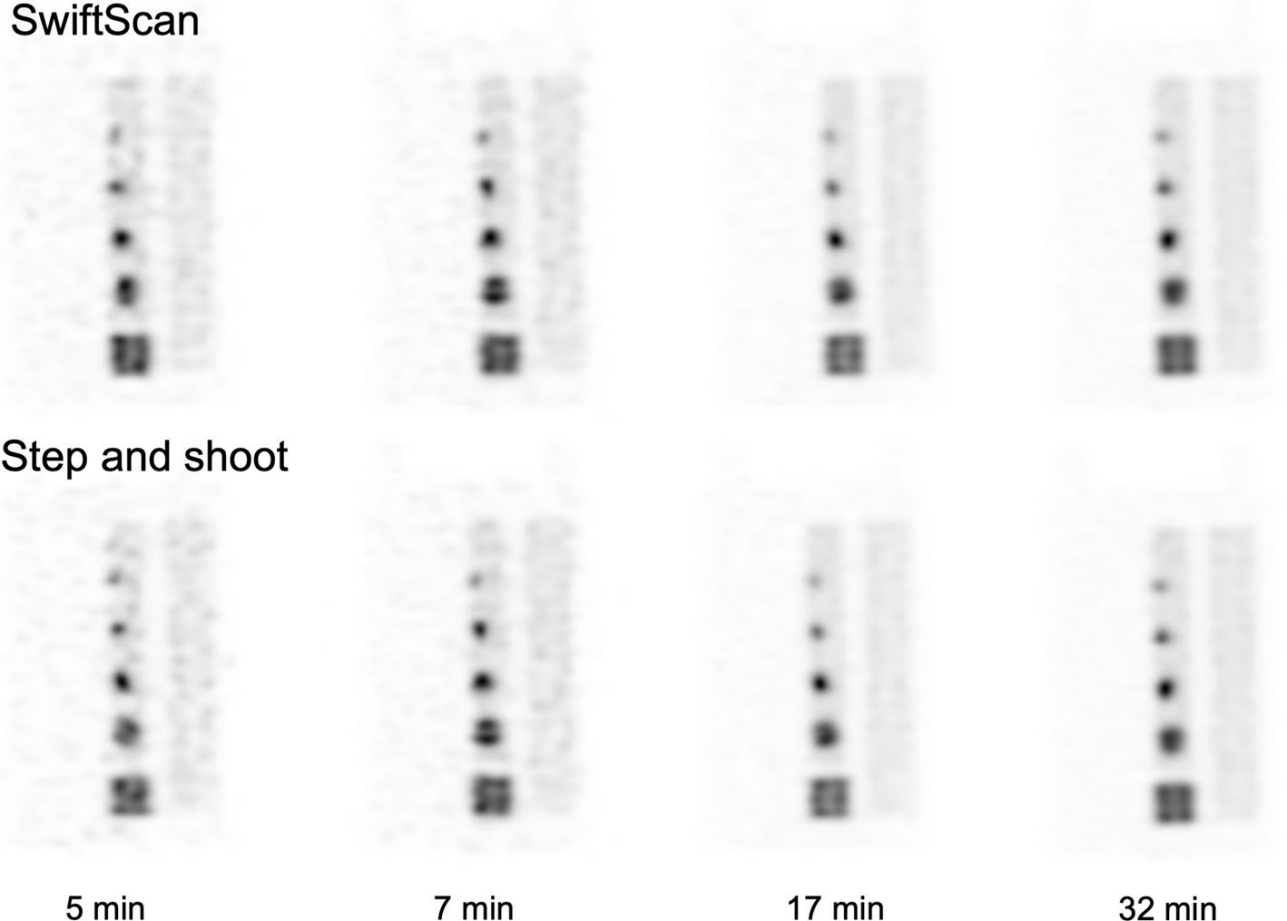
The phantom images of various acquisition time using SwiftScan SPECT and step and shoot mode (SSM). The upper row shows SwiftScan SPECT and the lower row shows SSM

Figure 6 shows the effects of scan time for SwiftScan SPECT and SSM counts in a 17-mm sphere. The mean counts of both SwiftScan SPECT and SSM increased logarithmically with increasing examination time. The mean counts of SwiftScan SPECT were higher than those of SSM; the differences in mean counts between SwiftScan SPECT and SSM were 31.3%, 21.2%, 9.3%, and 1.8% for 17-mm spheres at acquisition times of 5, 7, 17, and 32 min, respectively. The trend was similar for other tumor sphere sizes.

### FWHM

**Fig. 7:**
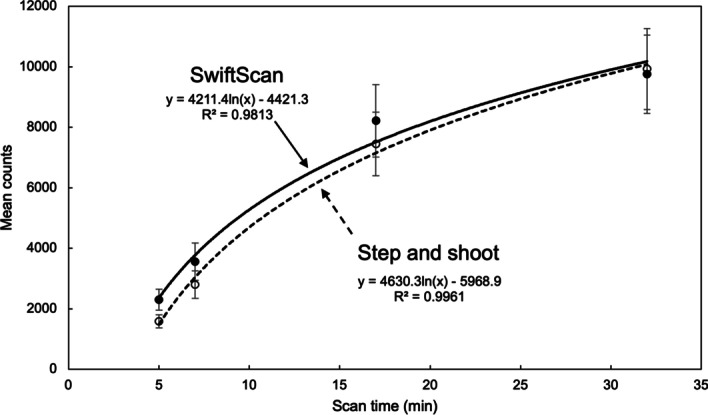
Relationships between scan time and mean counts. The solid line indicates SwiftScan SPECT and the dashed line indicates step and shoot mode (SSM). Error bar shows standard deviation

The line profile curves are illustrated in Fig. 7. In both methods, the amplitude of the background part was higher in the short-time scan than in the longtime scan (Fig. 7a, b), and this trend was more pronounced for SSM (Fig. 7b). A comparison of FWHM for SwiftScan and SSM is shown in Table 1. The FWHM for SwiftScan SPECT (13.6–14.8 mm) and SSM (13.5–14.4 mm) was similar regardless of scan time. Both acquisition methods showed a tendency for the variability in FWHM to increase as the scan time decreased.

### SwiftScan SPECT and SSM images

**Fig. 8:**
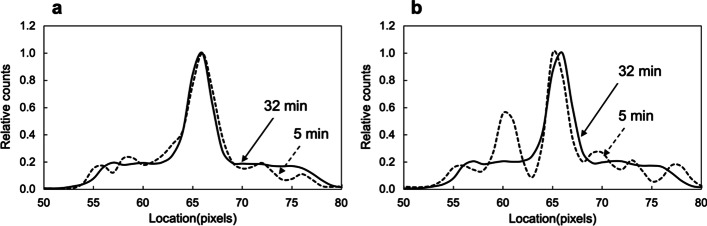
Comparison of line profile curves between short-time and longtime scans for **a** SwiftScan SPECT and **b** step and shoot mode (SSM). The solid line indicates a longtime scan (5 min) and the dashed line indicates a short-time scan (32 min)

Figure 8 shows the phantom images for each scan time. In both acquisition methods, noise was noticeable at short-time scan; however, the image quality improved as the scan time increased. There was no visually evident difference between the phantom images obtained by SwiftScan SPECT and SSM over 7-min scan time. However, the uniformity of the tumor and background area seemed slightly different between SwiftScan SPECT and SSM at 5-min scan time.

### Accuracy of quantitative value

**Fig. 9:**
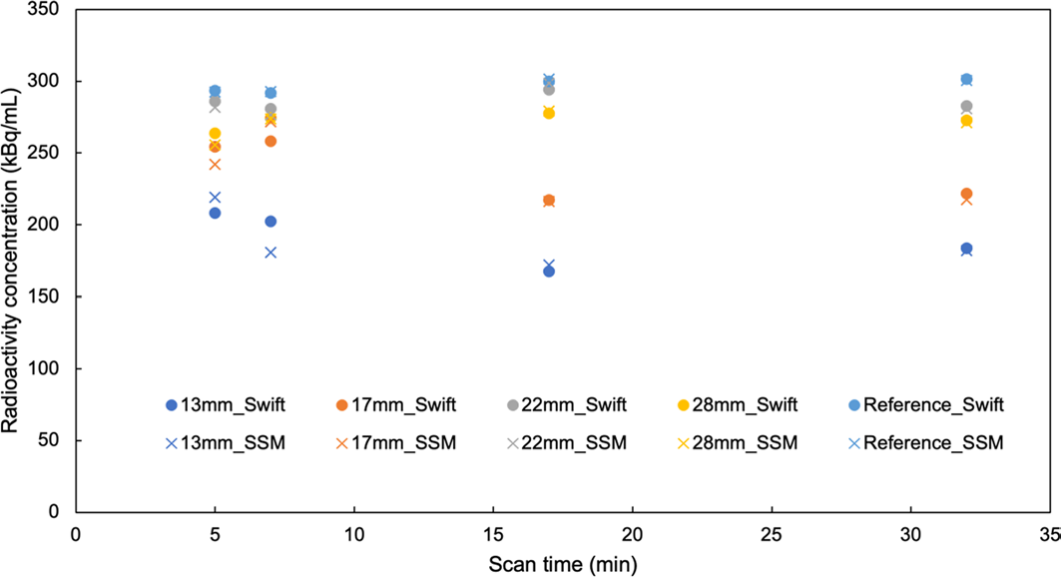
Radioactivity concentration for each diameter of tumor bone area at various scan times. Circles indicate SwiftScan SPECT and cross marks indicate step and shoot mode (SSM)

**Fig. 10:**
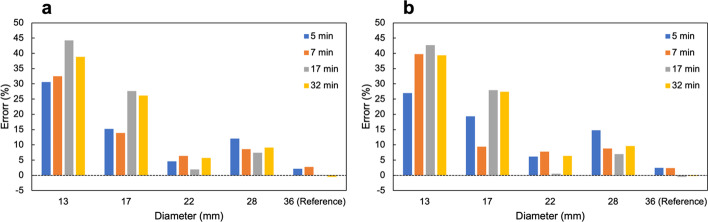
Error per scan time in quantitative values at each tumor diameter for **a** SwiftScan SPECT and **b** step and shoot mode (SSM)

Figure 9 shows the relationship between measured mean radioactivity concentration for each diameter of tumor bone area and acquisition times. The quantitative value of SwiftScan SPECT and SSM was similar; the measured mean radioactivity concentration of both SwiftScan SPECT and SSM decreased as the diameter of the tumor area decreased. In the small tumor diameter (e.g., 13 and 17 mm), the short-time scan showed a tendency of higher radioactivity concentration than that of longtime scan. Figure 10 shows the measured radioactivity concentration errors. The errors of quantitative values in 17-mm spheres at acquisition times of 5, 7, 17, and 32 min were 15.2%, 13.9%, 27.6%, and 26.1%, respectively, for SwiftScan SPECT and 19.3%, 9.4%, 28.0%, and 27.4%, respectively, for SSM. Both methods indicated an increasing error as tumor diameter size decreased; above 22 mm, the short-time scan showed similar or larger errors than the longtime scan, whereas at 13 and 17 mm, the short-time scan showed more minor errors. The MAE of measured radioactivity concentration for SwiftScan SPECT and SSM was as follows: 5 min, 38.8 and 41.8; 7 min, 38.4 and 40.8; 17 min, 48.8 and 47.2; and 32 min, 48.1 and 49.8 (Table 1).

## Discussion

This study investigated the effects of scan time for SwiftScan in quantitative bone SPECT using a phantom. SwiftScan SPECT showed a trend toward superiority over SSM in count statistics and image quality in short-time scans. In addition, SwiftScan SPECT was similar to or better than SSM in quantification regardless of the scan time. These results suggest that SwiftScan SPECT provides the same or better image quality and quantification as SSM in short scan time quantitative bone SPECT examination.

The mean SPECT counts of SwiftScan SPECT tended to be higher than those of SSM for short-time scan. In addition, the CV of SwiftScan SPECT was lower than that of SSM for short-time scan, but the CVs of both methods were equivocal for longtime scan. This could be because SwiftScan SPECT adds the count of detector rotation time (approximately 4 s/view, total 120 s) to the SSM. When the scan time was 5 min, SwiftScan SPECT increased the acquisition time by 1.67 times (6 s/view + 4 s/view) compared with SSM (6 s/view), whereas when the scan time was 32 min, SwiftScan SPECT increased the acquisition time by approximately only 1.07 times (60 s/view + 4 s/view) compared with SSM (60 s/view). Cao et al. [19] compared the image quality of simulated continuous SSM (CSSM) with that of SSM through simulation experiments and reported that CSSM was comparable to SSM for longtime acquisition, but for short-time acquisition, CSSM was superior to SSM as the image quality was substantially improved. SwiftScan SPECT also includes blurring while the detector moves to the next acquisition position. Therefore, it is assumed that the CV of SwiftScan SPECT reflects the increase in count statistics and the blurring effect resulting from the detector movement.

The CNR of SwiftScan SPECT was higher than that of SSM in short-time scan. However, the CNR of SSM was similar to or higher than that of SwiftScan SPECT in longtime scan. This finding could be attributed to the same reason as described above for CV: In the short-time acquisition, the effect of the added counts during detector movement in SwiftScan was significant. The acquisition parameters, such as the number of projections and acquisition time, are less relevant in regions with high-count statistics (e.g., in the tumor area) compared with regions with low-count statistics (e.g., in the background) [13]. The extended acquisition time per view of the SwiftScan SPECT compared with the SSM would result in suppressed background noise, thereby improving CNR. Therefore, SwiftScan SPECT is more advantageous than SSM for short-time scans because the image quality can be improved by summing up the counts during the detector rotation time. However, for longtime scans, there might be no advantage in using SwiftScan SPECT instead of SSM.

In the FWHM evaluation, although SwiftScan SPECT showed a slightly higher FWHM at all scan times than SSM, the difference was 0.1–1.2 mm and was not significant. In quantitative bone SPECT, spatial resolution is an important factor because it can reduce the underestimation of small tumor bone uptake due to the partial volume effect [24]. Matsutomo et al. [25] reported no difference in FWHM between continuous repetitive rotation acquisition and SSM. Our results are similar to previous study results. A tendency toward more significant variability in FWHM was observed for both methods when the scan time was short. This trend was strongly influenced by background noise as shown in the line profiles. In particular, a divergence was noted between long and short scan times in the line profiles of SSM compared with SwiftScan SPECT. As with CV, the profile curves for SwiftScan SPECT might reflect the effects of increased count statistics and blur due to the movement of the detector.

To assess the convergence of the radioactivity concentrations for short- and longtime acquisitions, we fixed the reconstructions to 10 subsets and varied the iterations. The results showed that the mean radioactivity concentrations of SSM and SwiftScan SPECT converged at more than five iterations. No difference in the convergence between the two acquisition methods was observed during the longtime acquisition. Interestingly, the mean radioactivity concentration of SwiftScan tended to be closer to the actual value for short-time acquisition. Furthermore, with an increasing number of iterations, the maximum concentration of radioactivity of SwiftScan SPECT tended to be close to the actual radioactivity concentration. This finding could be attributed to the superiority of SwiftScan SPECT over SSM in count statistics, as the variation of the maximum and mean radioactivity concentration with the number of iterations in the SwiftScan SPECT short-time acquisition showed a similar trend to that in the longtime acquisition. A previous study reported that the convergence of SPECT with a tumor-to-background ratio of 9.7:1 and an acquisition time of 20 s per projection converged with an iteration number of 9 on hot spheres larger than 17 mm [26]. The authors of that study also found that using a higher than optimum number of iterations resulted in overestimation of and/or variation in the maximum radioactivity concentrations. In our study, we set the number of iterations for a 28-mm sphere based on the previous study [10]. It should be noted that the optimal number of iterations may be influenced by the noise level and size of the ROI; thus, further research is warranted.

SwiftScan SPECT and SSM were similar in quantitative value comparison regardless of scan time. A previous study has reported that SwiftScan SPECT does not affect quantification in clinical bone quantitative SPECT, even with 25% less acquisition time than SSM [20]. These results support the use of SwiftScan SPECT to reduce examination time or injection radioactivity. Conversely, in both SwiftScan SPECT and SSM, the measured mean radioactivity concentration of small diameter (13 and 17 mm) for the short-time scan was higher than that of the longtime scan. The reconstruction packages with resolution correction based on collimator–detector response [27] that we have used have been found to produce edge and oscillation artifacts on spheres with homogeneous radioactivity distribution [28]. When sphere size decreases, the edge artifacts come very close to each other and eventually merge, resulting in considerably high activity in the center of the sphere [27–29]. The quantitative analysis software accompanying the SPECT/CT system used in this study was designed to calculate quantitative values without noise reduction filters. Therefore, the noise reduction filter was not used for all SPECT images. The quantitative values of a small sphere in a short-time scan may have been overestimated due to the extremely high noise level and the effect of edge artifact in the VOI. This might have led to an underestimation of the quantitative value error for short-time acquisitions and smaller MAEs for short-time acquisitions than for longtime acquisitions. Collarino et al. [26] reported that changing the threshold value when setting the VOI using the isocontour method altered the quantitative value. The setting of the VOI affects the quantitative values, and further studies are needed to achieve accurate and reproducible quantitative values.

In quantitative bone SPECT, attempts to reduce acquisition time are being investigated. Ichikawa et al. [30] showed that the 3-min acquired SPECT with ordered subset conjugate gradient minimizer reconstruction algorithm could maintain equivocal image quality and quantification as a 9-min acquisition. Although the reconstruction algorithm currently depends on the vendor, there is a possibility of further reducing the acquisition time and/or improving image quality and quantification by applying a more advanced reconstruction algorithm to SwiftScan SPECT. Moreover, SwiftScan SPECT may reduce the total examination time if the image quality and quantification are sufficient. Reducing the examination time and obtaining quantification and image quality comparable to that of SSM would reduce the possibility of patient motion and mental and physical distress.

This study has several limitations. First, the effect of the change in the acquisition time and radioactive concentration level was not investigated in detail because it was a phantom experiment. Therefore, it is necessary to investigate the detailed acquisition time variation by simulation. Second, no comparison with the CM acquisition was performed. Because continuous acquisition is used in many SPECT/CT devices, a comparison is necessary. Lastly, there is a lack of clinical study. It is necessary to examine whether the results of phantom experiments are equivalent to those in clinical practice.

## Conclusions

SwiftScan in quantitative bone SPECT showed similar to or better quantitative performance than the SSM. Furthermore, the count statistics of SwiftScan SPECT were superior to those of SSM, improving the image quality. However, the improvement in image quality appears to be limited to short-time scans. Therefore, using SwiftScan SPECT instead of the short-time scan SSM scanning protocol in clinical practice might provide higher-quality diagnostic images. The results of this study could provide important information for using SwiftScan for quantitative bone SPECT.

## Data Availability

SPECT and CT raw datasets are available from the corresponding author on reasonable request. All data analyzed during this study are included in this published article.

## Abbreviations

SPECT: Single-photon emission computed tomography
SSM: Step and shoot mode
CM: Continuous mode
CSSM: Continuous step and shoot mode
LEHR: Low-energy high-resolution
LEHRS: Low-energy high-resolution sensitivity
CV: Coefficient of variation
CNR: Contrast-to-noise ratio
CT: Computed tomography
FWHM: Full width at half maximum
RC: Recovery coefficient
SUV: Standardized uptake value
AC: Attenuation correction
SC: Scatter correction
SPECT/CT: Single-photon emission computed tomography/computed tomography
OSEM: Ordered subset expectation maximization
ROI: Region of interest
VOI: Volume of interest
MAE: Mean absolute error
